# ECG R-wave peaks marking with simultaneously recorded continuous blood pressure

**DOI:** 10.1371/journal.pone.0214443

**Published:** 2019-03-28

**Authors:** Qiong Yu, Aili Liu, Tiebing Liu, Yuwei Mao, Wei Chen, Hongxing Liu

**Affiliations:** 1 School of Electronic Science and Engineering, Nanjing University, Xianlin Campus, Nanjing, China; 2 Nanjing General Hospital of Nanjing Military command, Nanjing, China; University of Minnesota, UNITED STATES

## Abstract

**Background and objectives:**

ECG signal is relatively weak and vulnerable to various noise interferences, such as electromyography. There will be robustness problems when detecting the instantaneous heart rate independently. In some cases, multiple human physiologic parameters are monitored to help in heart rate detection.

**Methods:**

In this paper, an algorithm that marks the R-wave peaks with the help of simultaneously recorded continuous blood pressure is proposed and tested on two databases. One database, called the challenge database, is provided by the PhysioNet/Computing in Cardiology Challenge 2014, and the other is the MGH/MF waveform database.

**Results:**

The final scores of the proposed algorithm are 97.3% for the challenge database and 96.6% for the MGH/MF waveform database.

**Conclusions:**

The experimental results show that this algorithm has high detection accuracy and a relatively strong robustness.

## 1. Introduction

Long-term and continuous monitoring of the instantaneous heart rate is the main means of human care [1] [2]. A common method to obtain the instantaneous heart rate is to decide the RR intervals of an ECG signal, making the key problem the detection of R-wave peaks [3].

A variety of algorithms have been applied to detect the R-wave peaks of an ECG. Generally, all these algorithms can be divided into three steps: (1) signal preprocessing, which improves the signal-to-noise ratio (SNR) before detection; (2) determination of the R-wave peak positions with the enhanced signal from the step (1); and (3) local searching for the accurate R-wave peak locations on the original waveform, with the help of the determined R-wave peak positions on the enhanced signal. For step (1), the classical methods of preprocessing are like the difference algorithm [4], Hilbert transform algorithm[5], template matching algorithm[6] and wavelet transform algorithm[7], etc.; for step (2), various algorithms have been proposed to set the proper threshold [8] [9] and conduct a local search.

When detecting R-waves during continuous long-term monitoring, even a sensational algorithm may end up with an unfavorable result, which may contain incorrect peaks and missed peaks due to various noise interferences, such as EMG (Electromyography) and respiration. Therefore, further enhancement of robustness is needed [7].

Multiple examples of human physiologic parameter monitoring exist in real life. In the process of some heart surgeries and other major operations, various signals are monitored, including the ECG signal, continuous and ambulatory blood pressure (BP) signal, continuous and dynamic oximetry (SO2) signal, respiratory signal (Resp), electroencephalogram (EEG) signal and other physiological parameters [10]. We should improve the robustness of the R-wave detection with the help of other physiologic signals since they are recorded simultaneously.

This idea has great importance both at home and abroad. In 2014, PhysioNet held a competition named the “Robust Detection of Heart Beats in Multimodal Data: the PhysioNet/Computing in Cardiology Challenge 2014” to encourage the exploration of robust methods for locating heart beats in continuous long-term data from bedside monitors and similar devices that record multiple physiological parameters (https://www.physionet.org/physiobank/database/challenge/2014/). Based on the above background, because of the relevance and synchronicity between the ECG signal and BP signal [11], we proposed an algorithm that detects the R-wave peaks with the help of the continuous BP signal recorded simultaneously. The test data are from the competition database and another database containing multiple physiological signals, called the MGH/MF waveform database (https://www.physionet.org/physiobank/database/mghdb/), which contains arterial pressure signals.

## 2. Methods

### 2.1Data description

The challenge databases used as our test data were the PhysioNet/Computing 2014 Cardiology Challenge and the MGH/MF waveform database.

In the challenge, 200 records were given to the competitors for the training data, each containing an ECG signal, continuous and ambulatory BP signal, EEG signal, and 4 to 8 other physiological signals. The sampling frequency was 250 Hz or 360 Hz, though in any given record, all signals were sampled at the same fixed frequency. The record duration was no more than 10 minutes, and the overall length was completely used for our test. The correct reference detection result of each record was given in its corresponding annotation (.atr) file [12].

The MGH/MF waveform database consists of recordings from 250 patients, and individual recordings vary in length from 12 to 86 minutes (in our test, only the first 10 minutes of each record are used). Typical recording includes three ECG leads, arterial pressure, pulmonary arterial pressure, central venous pressure, respiratory impedance, and airway CO2 waveforms. Some recordings include the intra-cranial, left atrial, ventricular and/or intra-aortic-balloon pressure waveforms. The sampling frequency was 360 Hz. Each record includes an annotation (.ari) file, which contains the beat and event labels [13].

### 2.2Proposed training algorithm

Before the main detection algorithm, introduction of a training stage is suggested. In real corresponding applications, it is reasonable and feasible to pre-monitor some people for a period of time to determine some individual parameters for the following long-time, regular detection, which is an important philosophy. The training algorithm proposed by us includes the following three aspects.

**Fig 1:**
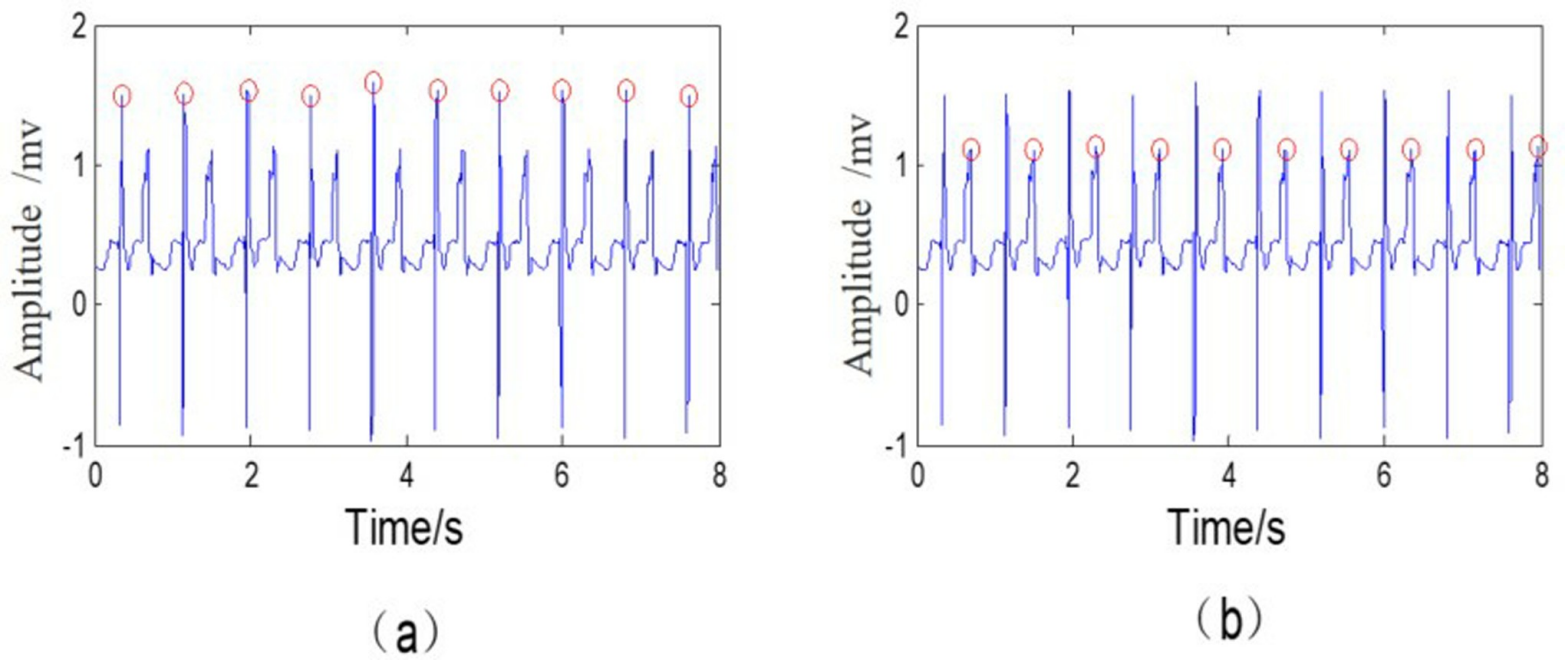
Wrong R-wave peaks detection in a pacemaker pattern. (a) Determine pacemaker pulses as R-wave peaks; (b) Correct detection of R-wave peaks.

First, an offline pacemaker pulse template library was formed. Compared to the regular ECG signal, a pacemaker ECG has pacemaker pulses with a narrow width and higher height, and it is easy to determine the pacemaker pulses as R-wave peaks, bringing about the false detection shown in Fig 1A, while the correct marks are shown in Fig 1B. Our solution is to first eliminate the pacemaker pulses based on the pacemaker pulse template library before the normal R-wave detection. To make the offline pacemaker pulse template library, we did the following: (1) for each pacemaker record, its pacemaker pulse peaks were marked with the help of the human eye, and (2) took 0.5*LEN before each pulse peak and 0.5*LEN after it as a pacemaker pulse complex (***LEN*** = **1*s***), then averaged all the cut out pulse complexes to form a pacemaker pulse template.

Second, the combination vector of features used in R-wave confirmation were trained. Usually, we need to judge the initial ECG R-wave detection result and modify it. The confirmation of an R wave true or false detection should be based on multiple features of the wave. The method of linear discriminant analysis (LDA) can be applied to train the combination vector on the multiple features, with which we can form a combined feature to judge the R wave detection result. In this paper, two features of the wave are defined, one is the waveform similarity with the obtained current R-wave template, defined by their correlation coefficient, and the other one is the normalized wave peak (or amplitude) value. The steps of the LDA-based combination vector training using the two features are: (1) to establish the training samples, calculating the two features of the truly detected R waves and falsely detected R waves in the database and marking their class labels; (2) to calculate the combination vector with the established training samples according to the LDA theory. In this paper, we obtained the values of the combination vector as [0.9303, 0.3667], where 0.9303 is for the correlation coefficient, and 0.3667 is for the normalized amplitude.

**Fig 2:**
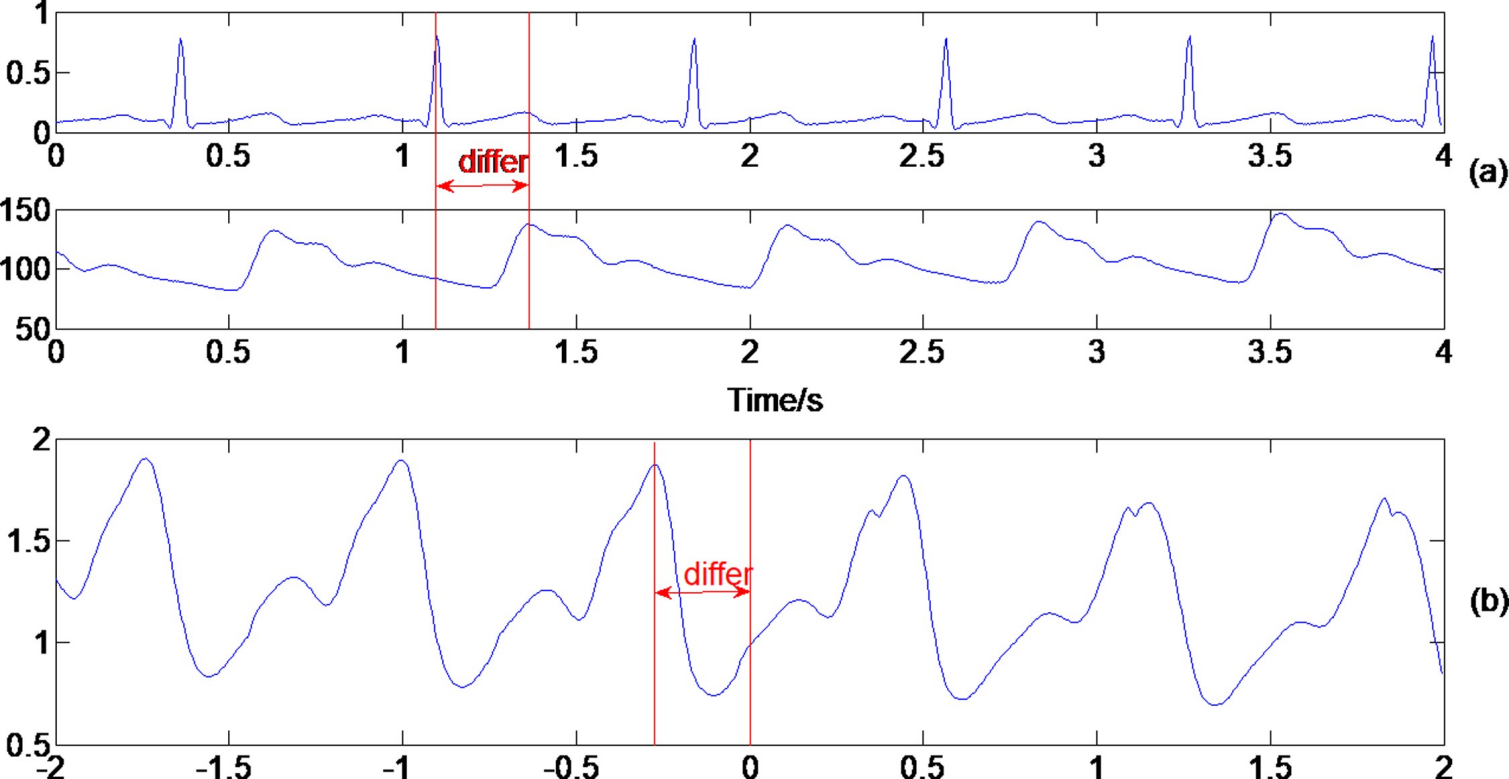
Determination of the time delay ‘differ’ between the corresponding peaks of the ECG and BP. (a) Illustration of the ‘differ’ between the ECG and continuous BP; (b) Cross-correlation function to determine the ‘differ’.

Third, the time delay between BP peak and corresponding ECG peak was trained, marked as ‘differ’ here and is shown in Fig 2A. As we hope improve the algorithm effectiveness, this delay parameter is the most important one to use in the fusion algorithm. The ‘differ’ time delay between two corresponding peaks of ECG and BP is obtained from the cross-correlation function, as shown in Fig 2B. Noticing, the quality of the pre-monitored ECG and BP signals for determining the ‘differ’ should be good enough. In this paper, we select the choice ECG and BP segment to calculate the cross-correlation function, and if there are pacemaker pulses on ECG, we should eliminate them first based on the built pacemaker pulse template library as in the later main detection algorithm. The basic choice ECG and BP segment selection algorithm is as follows:

(1) Initially detect peaks on each 12-s-length segments of ECG and BP, and make a waveform template for each of them respectively;
(2) For each ECG segment, get the cross-correlation coefficient of each detected R wave with the template signal, called *C*_*i*_ *i* = 1,…, *N*, N is the number of detected R peaks; then, set ‘copeak’ = median(*C*_*i*_ *i* = 1,…,*N*); similarly, calculate ‘cobp’ for each BP segment;
(3) Set the coefficient ‘coeff’ = ‘copeak’*’cobp’ for each segment. If the max value of all ‘coeff’s is higher than 0.8, the corresponding ECG and BP segments are taken as the choice ones to calculate the best ‘differ’. If not, set ‘differ’ to an empirical value (0.2 s) statistically.

### 2.3 Proposed detection algorithm

**Fig 3:**
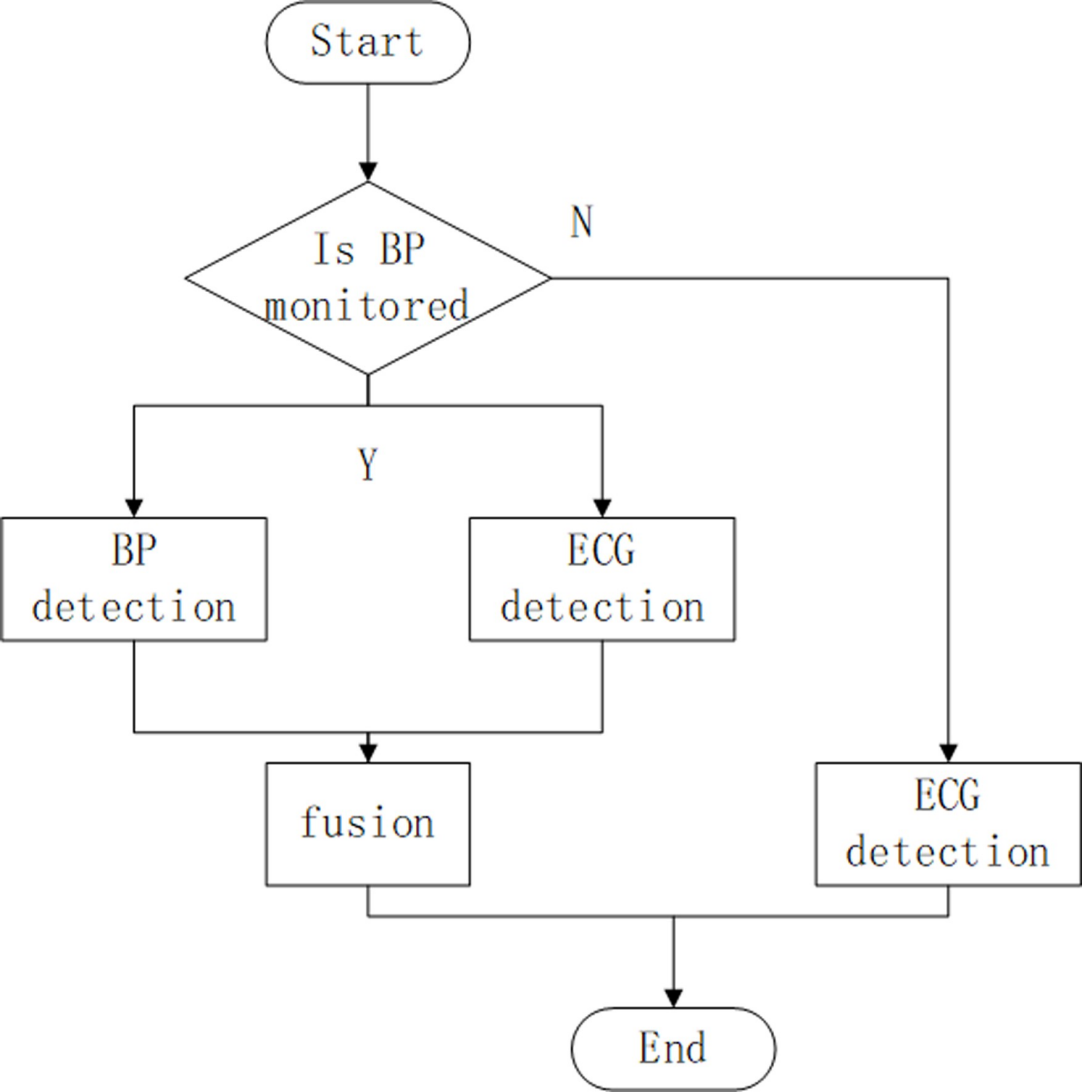
Overall block diagram for the proposed R-wave peak detection algorithm.

The overall block diagram for our proposed R-wave peak detection algorithm can be found in Fig 3.

The basic strategy behind the algorithm is that when BP is not monitored, we detected the ECG R-wave peaks directly and output them as the final results; on the contrary, the fusion of ECG and BP respective detection results were used as an output. First, a threshold algorithm was used to determine whether BP is monitored. Take the threshold ‘a’ = 10 and ‘b’ = 2. If the peak value is less than or equal to threshold ‘a’ or the mean value of its absolute value is less than threshold ‘b’, BP is regarded as not being monitored.

In the following subsections, three blocks, including the ECG detection, BP detection and fusion, will be thoroughly explained.

#### 2.3.1 ECG detection

**Fig 4:**
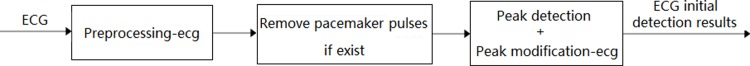
Flowchart of ECG detection.

The flowchart of ECG R-wave peak detection is shown in Fig 4.

**Fig 5:**
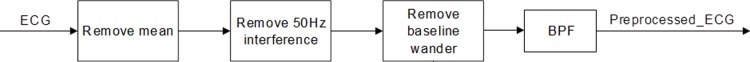
ECG preprocessing procedure (preprocessing-ecg).

**Preprocessing-ecg:** The ECG preprocessing procedure can be seen in Fig 5. To remove the 50 Hz interference, a notch filter (at 50 Hz) is used. To suppress the baseline wander, a two-order smooth filter (0.6 s window) is applied to ECG. Finally, a bandpass FIR filter between 0.5–80 Hz is used to further suppress the noise of the ECG.

**Fig 6:**
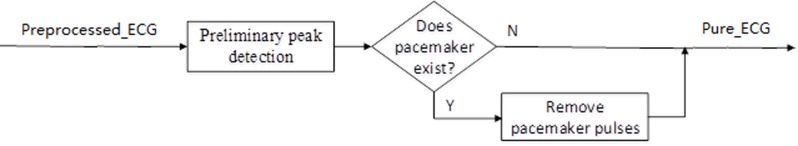
Flowchart for removing the pacemaker pulse.

**Remove pacemaker pulse if it exists:** The overall decision tree for removing pacemaker pulse is shown in Fig 6.

- Preliminary peak detection:
  This step aims to find all paced beats if they exist, and a peak detector based on the max search is applied (Sameni 2010 [14]). In this detector, the window length of max search is variable, and the smaller it is, the more peaks will be detected. After many tests, we set it to 0.2 s, which is small enough to detect all the possible pacemaker pulses.
  Before detecting the wave peaks, we cut the signal into segments, with lengths set to 24 s, and then performed the peak detection above on each segment separately.
- Determining whether the pacemaker pulse exists:
  To determine whether the pacemaker pulse exists, the specific procedures are as follows.
  1. Get the offline pacemaker pulse template library Template(i),i=1,⋯,M, which are made in advance. M is the number of templates in this library.
  2. Get the above preliminary peak detection results Rpeak(j),j=1,⋯,N, N is the number of detected R waves; then, do the cross-correlation between *Template* (*i*) and *Rpeak* (*j*), and then obtain *coeff* (*i*, *j*), *coeff* is an M*N matrix.
  3. For each row coeff(*i*,:), set Group(k)=coeff(i,(k-1)*10+1:k*10),k=1,⋯,floor(N/10). Then, set Gmax(k)=max(Group(k)); and finally, set me(i)=median(Gmax).
  4. After processing all the rows similarly, the M median values can be obtained, and the template that has the highest median value, named *P_template*, can be selected as the most correlated one. Lastly, set coef=max(me).
  5. If coef>0.9, it indicates that on the ECG signal, pacemaker pulses exist.
- Remove the pacemaker pulse when it exists:
  After determining the ECG signal pacemaker pattern, mark the peaks whose correlation coefficients with *P_template* are higher than 0.8, and then set their voltage to 0.

After determining the ECG signal pacemaker pattern, mark the peaks whose correlation coefficients with *P_template* are higher than 0.8, and then set their voltage to 0.

**Peak Detection:** The detection accuracy of the ECG signal will surely affect the final result, especially when BP is not monitored. Therefore, besides the ECG preprocessing method shown in Fig 5, we can use an additional preprocessing method called wavelet transformation to decrease the noise and interference. In this article, a CWT (continuous wavelet transform) was used. The wavelet basis is cmor1-1.5, and the wavelet scale is 120, when the sampling rate is 1000 Hz. When the sampling rate is ‘fs’, the wavelet scale calculation formula is as follows:

scale=120*fs/1000

After obtaining the enhanced signal using CWT, we also cut it into segments with lengths set to 24 s. Then, the peak detection will be performed on each segment separately.

The peak detection is also based on the peak detector (Sameni 2010). However, the differences from the detection of pacemaker pulses are: (1) the max search window length is set to 0.4 s; (2) a threshold is applied. The method of obtaining the threshold is: (1) cut the current detected segment into 10 shorter segments and get the maximal value of each shorter segment first; (2) the threshold is just 1/2 as much as the minimum of all the maximal values. The peaks whose values are higher than the threshold will remain.

**Peak modification-ecg:** The modification of the ECG peak detection result is based on pattern recognition. The main steps are: (1) extracting two features of each detected R-wave peak, one is the correlation coefficient with the ECG template (the template is made with R-wave peak detection results), and the other is the normalized peak amplitude; (2) using linear discriminant analysis (LDA) to combine the two features into a new one (the combination vector has been calculated in advance); (3) setting a threshold, and if the value of the new feature is higher than the threshold, the corresponding R-wave peak will remain; otherwise, it will be deleted.

Modification is performed on each segment whose duration is set to 24 s. When modifying, after calculating the feature combination value for each detected peak, the threshold for selecting the R-wave peaks is adapted. Try different thresholds (from small to large) and select the threshold that corresponds to the smallest standard deviation value of the RR intervals as the final threshold.

#### 2.3.2 BP detection

**Fig 7:**
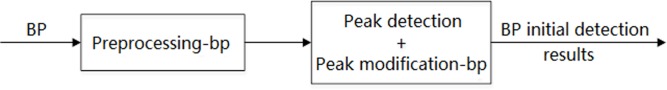
Flowchart of BP detection.

The flowchart of BP wave peak detection is shown in Fig 7.

**Fig 8:**
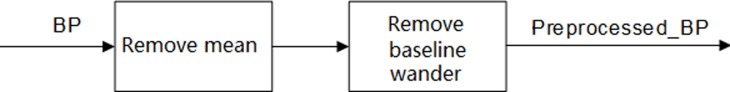
BP preprocessing procedure (preprocessing-bp).

**Preprocessing-bp:** The flowchart of BP preprocessing is shown in Fig 8. To suppress the baseline wander, a two-order smooth filter (2s window) is applied to the BP.

**Peak detection:** Implementing the same peak detection based on the max search and threshold as ECG on the preprocessed BP signal, the BP detection results can be obtained.

**Peak Modification-bp:** The BP modification is based on the template method due to its simple and relatively stable waveform.

(1) Make a BP complex template whose duration is set to 1 s.
(2) Get the correlation coefficient between each detected BP complex and the template signal.
(3) Compare each coefficient with 0.6: if it is lower than 0.6, the corresponding detected peak will be deleted from the result.

#### 2.3.3 Fusion

**Fig 9:**
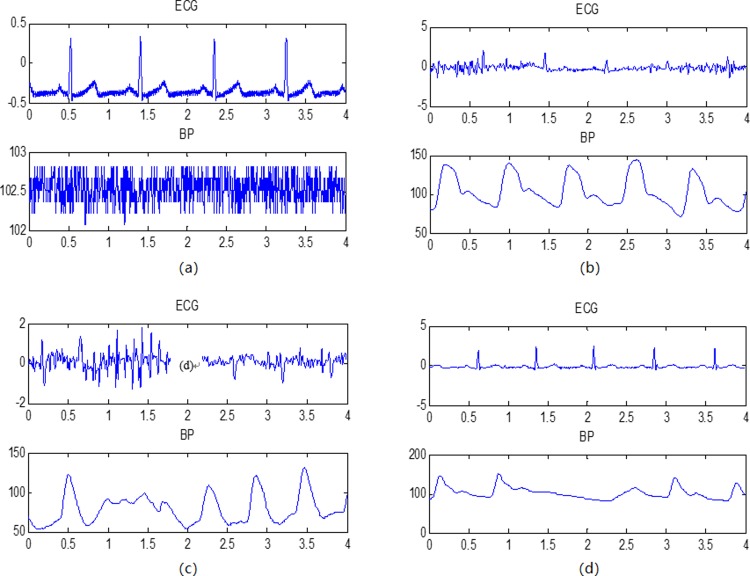
Different kinds of qualities on simultaneously recorded ECG and BP signals. (a) BP is not monitored; (b) (c) the BP waveform is better than the ECG waveform; (d) the ECG waveform is better.

**Fig 10:**
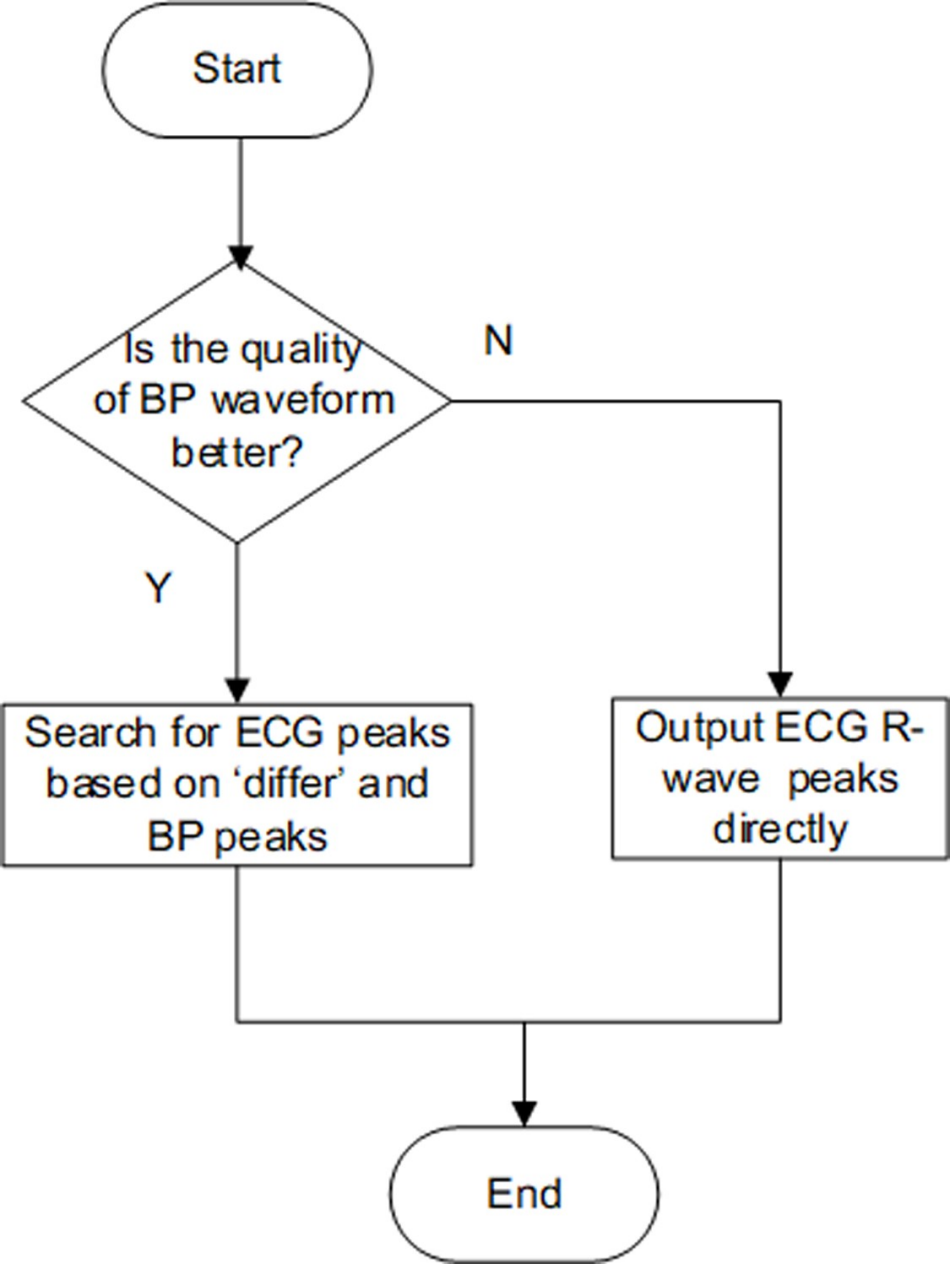
Flow diagram for fusion.

In this step, first cut the signals into shorter segments, and for each segment, when BP is monitored abnormally (Fig 9A), we can only output ECG initial detection results directly. When the BP waveform is better than the ECG waveform (Fig 9B and 9C), considering that the continuous BP signal features a simple waveform, which is easy-to-understand and shows periodic synchronization with ECG, the BP initial detection result can be used to help detect the ECG R-wave (taking the detected BP peak positions as a reference and searching for ECG R-wave peaks based on the ‘differ’ within the range of ‘thrm’, which is set to 0.1 s after a large number of tests). In contrast, when the ECG waveform is better (Fig 9D), we can ignore the BP and output ECG initial detection results directly. The flow diagram of the fusion can be seen in Fig 10.

**Compare the quality of ECG and BP:** The template matching algorithm is used to compare the quality of the BP waveform and ECG waveform on each segment. Segments that are too short will make it difficult to establish a waveform template, while segments that are too long will lower the performance of the fusion step. Thus, first, we cut the ECG and BP signal into segments of 24 s in length and made templates for each segment. Then, each segment was cut into shorter segments of 4s in length, and the quality of ECG and BP on each shorter segment was compared. The specific steps are as follows:

(1) Calculate the QRS template with the detected ECG R-wave peak positions on 24 s-long segments and the BP template similarly;
(2) Cut the current segment into shorter segments of 4 s in length. For each shorter segment, compare the quality of the ECG and BP: (a) get the cross-correlation coefficient of each detected R wave with the template signal, called *C*_*i*_ = (*i* = 1,…,*n*) (n is the number of detected ECG peaks on the current shorter segment); (b) calculate the ECG matching coefficient: $\text{R}{\text{e}}_{ecg}=median([C_{1},C_{2},\ldots ,C_{n}])$; (c) similarly, obtain the BP matching coefficient Re*_bp_*; (d) compare the matching coefficients of BP and ECG: Re_bp_ and Re_ecg_, and if ${\text{Re}}_{\text{bp}}\geq {\text{Re}}_{\text{ecg}}$, this indicates that the quality of BP is better than ECG for this segment. Otherwise, it shows that the quality of ECG is better.
(3) For each shorter segment, output the detection results of the signal with better quality.

### 2.4 A special step for the MGH/MF waveform database

The above algorithm is based on the condition of one ECG and one BP signal. However, there are 3 ECG signal leads for each record of the MGH/MF waveform database, so a special step is added when testing on this database.

This step, based on the template matching method, is used to pick out the best-quality ECG signal from the three ECG leads. For each ECG signal, we calculated its quality index name ‘Reecg’ and chose the lead with the highest ‘Reecg’ for further processing. The steps for calculating ‘Reecg’ are as follows:

(1) Detect the R-wave peaks using the peak detector above, and make a 0.3-s-length QRS template;
(2) Get the cross-correlation coefficient of each detected R wave with the template signal, called *C*_*i*_ (*i* = 1,…,*N*); N is the number of detected R peaks;
(3) For i = 1: N, if *C*_*i*_>th, T = T+1, endif, endfor, (th = 0.7);
(4) Calculate the ECG quality index: Reecg = *T* / *N*.

## 3. Test and score

First, we tested the algorithm with the 200 training records from the challenge database given by the official website and then compared this with the official ‘qrs’ marks from the ‘.atr’ files to get the final score. The details about the scoring rules can be found in Silva et al. [12]. In addition, Se (Sensitivity) = TP/(TP+FN) and PPV (Positive Predictivity) = TP/(FP+TP), where TP, FN, and FP represent the true positive (i.e., correct detected QRS), false negative (i.e., missed QRS) and false positive (i.e., extra detected QRS), respectively. Table 1 shows the results of the improved algorithm proposed in this paper and the recently published algorithms. The overall score of our algorithm is between the 1^st^ of Urška Pangerc et al.[15] and the 2^nd^ of Alistair E W Johnson et al.[16].

**Table 1:** Results of different techniques on the updated challenge training database.

Algorithm	Num. of Records	SE (%)						PPV (%)						Score (%)
		Min	Max	Ave.	σ	Gross	H	Min	Max	Ave.	σ	Gross	H	
Urška Pangerc et al.	200	-	-	97.8	-	98.1	-	-	-	97.2	-	97.5	-	**97.6**
Alistair E W Johnson et al.	200	-	-	96.5	-	96.9	-	-	-	95.1	-	94.2	-	**95.6**
M-code sample entry	200	-	-	84.0	-	84.3	-	-	-	82.3	-	80.9	-	**82.9**
This paper for all Recordings	200	37.6	100	96.5	7.23	96.7	-	40.1	100	97.8	6.84	98.3	-	**97.3**
This paper for Recordings with Pacemaker	10	86.0	100	96.3	5.03	96.4	0	86.1	99.9	97.0	4.71	97.1	0	**96.7**
This paper for Recordings without Pacemaker	190	37.6	100	96.6	7.34	96.7	0	40.1	100	97.8	6.94	98.3	0	**97.4**

Note: σmeans standard deviation, H means the result of the T-test, and them in tables below mean the same.

Similarly, we tested the algorithm with the MGH/MF waveform database and compared the score with that of Urška Pangerc et al. The overall comparative results are shown in Table 2.

**Table 2:** Results of different techniques on the MGH/MF waveform database.

Algorithm	Num. of Records	SE (%)						PPV (%)						Score (%)
		Min	Max	Ave.	σ	Gross	H	Min	Max	Ave.	σ	Gross	H	
Urška Pangerc et al.	247	-	-	95.9	-	96.5	-	-	-	93.9	-	94.1	-	**95.1**
This paper for all Recordings	247	42.3	100	96.2	6.04	96.3	-	25.5	100	96.9	8.97	97.0	-	**96.6**
This paper for Recordings with Pacemaker	12	88.7	99.0	95.8	3.37	96.1	0	86.4	100	97.5	3.98	97.5	0	**96.7**
This paper for Recordings without Pacemaker	235	42.3	100	96.2	6.15	96.3	0	25.5	100	96.9	9.15	97.0	0	**96.6**

The comparative scores show that our algorithm is superior to most competitors and comparable to the team (Urška Pangerc et al.) with the highest score.

**Fig 11:**
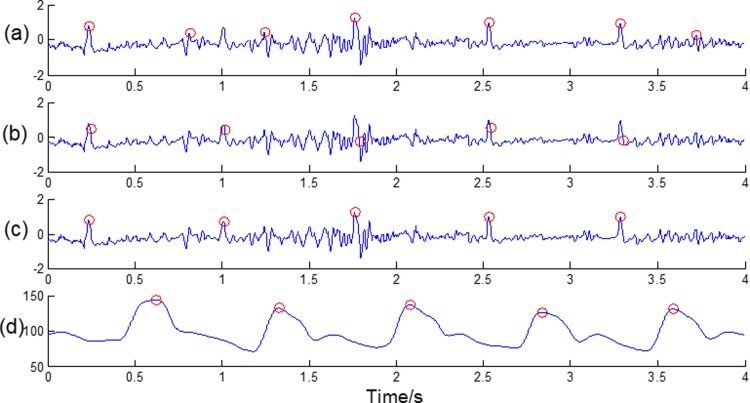
Comparison of detecting R-wave peaks independently and with the help of the BP signal. (a) Marks of the ECG R-wave peaks, which were detected independently. (b) Official marks of the ECG peaks. (c) Marks of the ECG peaks using our proposed fusion algorithm. (d) Marks of the BP peaks by our proposed fusion algorithm.

**Fig 12:**
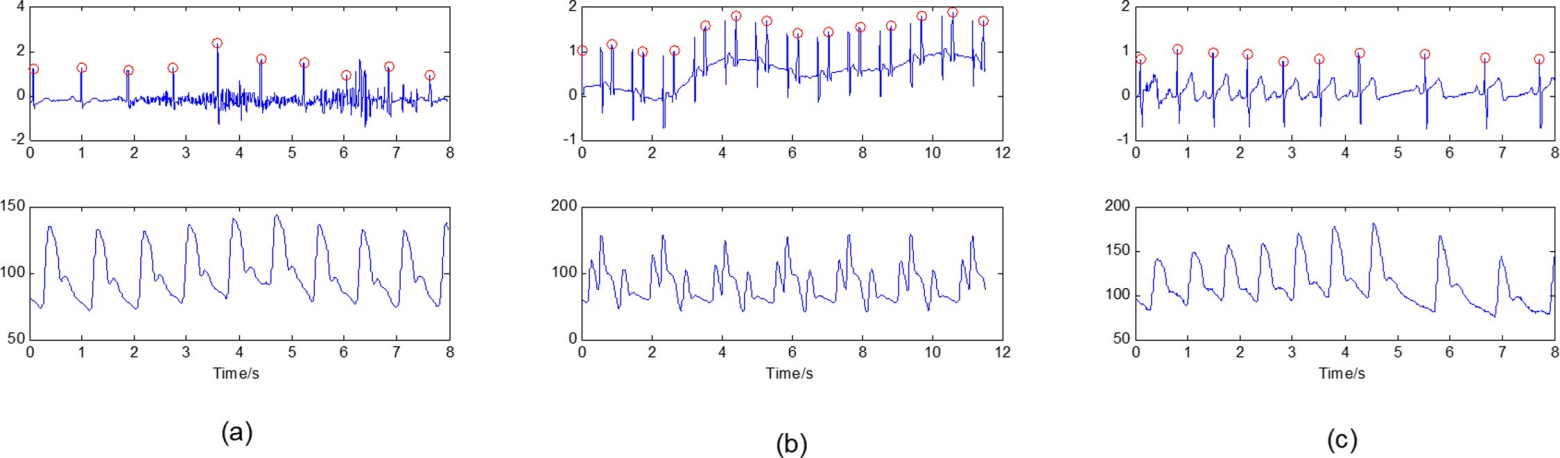
Successfully detecting the R-wave peaks in different patterns using our proposed algorithm. (a) A record with noisy ECG signal; (b) A record with pacemaker; (c) A record with a sudden change of RR-intervals.

Fig 11 shows our proposed fusion algorithm’s effectiveness. We can see that when the ECG waveform is in poor quality for noise interference, our fusion algorithm avoids some false and redundant detections and gets a better result. Fig 12 gives some other examples showing our algorithm’s effectiveness in detecting R-wave peaks.

## 4. Discussion

From the test results, it has been shown that the detection accuracy and robustness of the proposed algorithm are both superior to the most competitors’ algorithms and very competitive with that of the best algorithm. Although fusion strategies are commonly used in the competitors’ works in the PhysioNet/Computing 2014 Cardiology Challenge [15, 16], the fusion method proposed in this manuscript is different and seems more efficient. We also think that there are two main factors contributing to our algorithm’s effectiveness.

First is the setup of the offline pacemaker pulse template library in the training stage or pre-monitoring stage, which allows for the pacemaker pulses on the ECG to be detected and eliminated efficiently. From above Tables 1 and 2, we can know that the performance score of our algorithm is similar for recordings with and without artificial pacemaker pulses. We did T-test (α = 0.05) between recordings with artificial pacemaker and all recordings, also T-test (α = 0.05) between recordings without artificial pacemaker and all recordings, whose results are shown in Tables 1 and 2, indicating that our method has a good robustness to whether a data has pacemaker pulses or not.

Second is the R-wave judgment method. In the method, two features, excluding the RR intervals, are used, and their combination vector is determined using LDA at the training stage, which can improve the detection accuracy robustly. Since we do not use RR interval uniformity as a feature, the judgment method has good robustness in case of sudden RR-interval change. As seen in Fig 12C, when the RR-intervals show a sudden change, our algorithm still did not lead to false marks.

**Fig 13:**
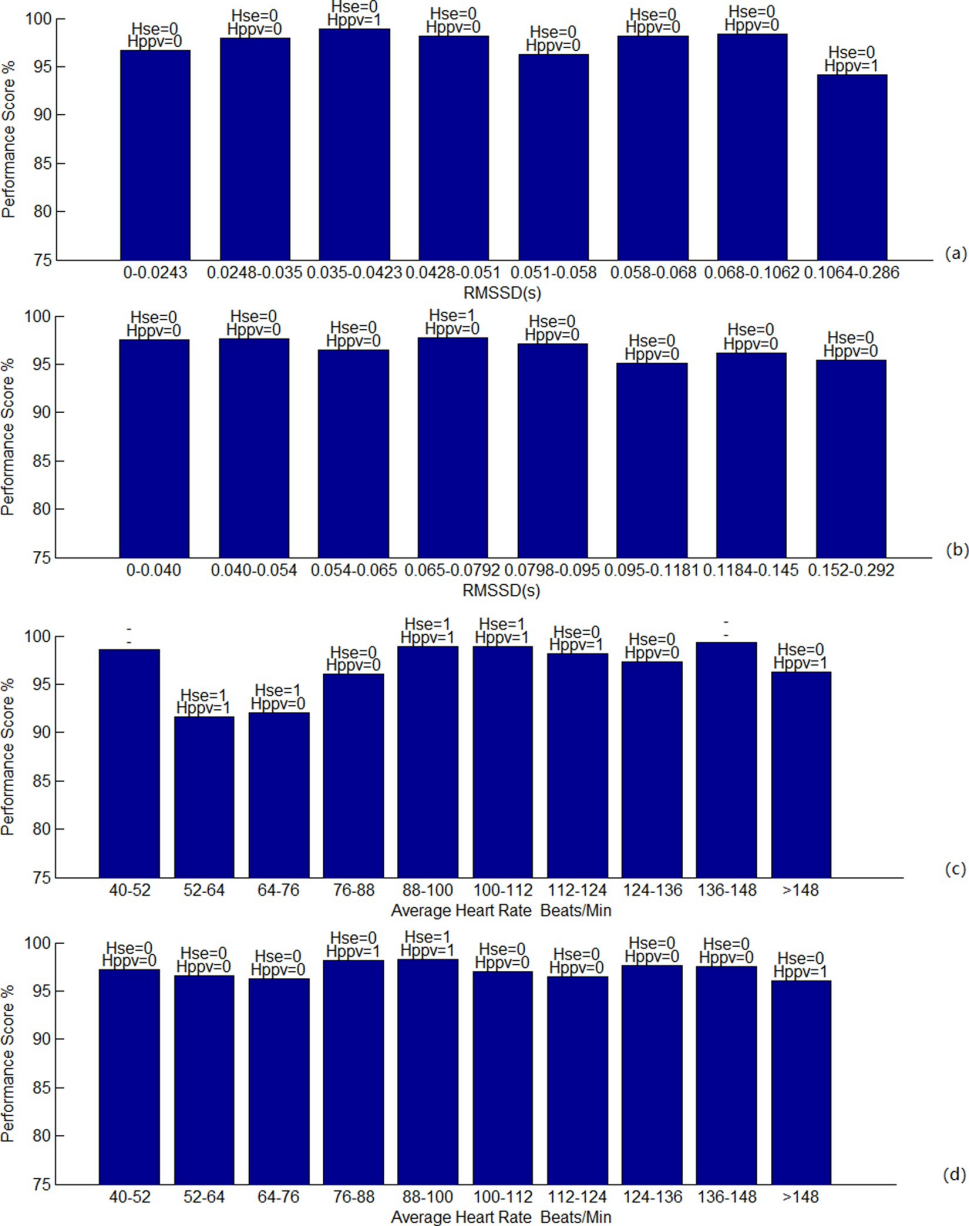
**Performance scores of our proposed algorithm under different heart rate variabilities and average heart rates**: (a) Different HRV for Challenge database; (b) Different HRV for MGH/MF database; (c) Different average heart rate for Challenge database; (d) Different average heart rate for MGH/MF database.

Further, we tested the independence of our algorithm from sudden changes in heart rate using all data in each database. We defined the heart rate variability of each data as its RMSSD, and assigned every data properly into one of 8 equal-number groups divided by RMSSD. After calculating the performance scores SE and PPV for each data, we statistically obtained their minimum, maximum, average and standard deviation σ, and the gross and overall performances for each divided group. After that, a T-test (α = 0.05) was performed on Se and PPV of each group of data and them of all data. Tables 3 and 4 have listed the tested results. Fig 13A and 13B are the graphs showing the overall performance score versus heart rate variability for two databases respectively.

**Table 3:** Performance scores of challenge database grouped by RMSSD.

RMSSD (s)	Num. of Records	SE (%)						PPV (%)						Score (%)
		Min	Max	Ave.	σ	Gross	H	Min	Max	Ave.	σ	Gross	H	
0–0.0243	25	37.6	100	94.7	13.2	95.8	0	40.1	100	97.1	11.93	98.9	0	**96.6**
0.0248–0.035	25	67.7	100	97.3	6.82	97.4	0	69.6	100	98.4	6.04	98.6	0	**97.9**
0.035–0.0423	25	85.0	100	98.9	3.00	98.8	1	82.1	100	99.0	3.52	99.0	0	**98.9**
0.0428–0.051	25	84.0	100	98.0	3.84	98.0	0	84.0	100	98.3	3.87	98.3	0	**98.2**
0.051–0.058	25	63.4	100	96.2	8.36	96.2	0	55.7	100	96.5	9.75	96.2	0	**96.3**
0.058–0.0686	25	83.7	100	97.3	4.12	97.1	0	83.1	100	98.7	3.48	99.3	0	**98.1**
0.0686–0.1062	25	80.8	100	97.8	4.00	97.7	0	80.9	99.9	98.9	3.76	99.1	0	**98.4**
0.1064–0.286	25	75.7	100	92.1	7.26	92.4	1	73.2	100	95.5	6.96	96.5	0	**94.1**

**Table 4:** Performance scores of MGH/MF waveform database grouped by RMSSD.

RMSSD (s)	Num. of Records	SE (%)						PPV (%)						Score (%)
		Min	Max	Ave.	σ	Gross	H	Min	Max	Ave.	σ	Gross	H	
0–0.040	31	67.2	100	97.7	6.02	97.3	0	64.3	100	97.1	6.81	97.8	0	**97.5**
0.040–0.054	31	70.4	99.7	97.2	5.31	97.3	0	79.8	100	97.8	4.95	98.1	0	**97.6**
0.054–0.065	31	42.3	100	96.0	10.26	95.6	0	44.8	100	97.1	10.01	97.1	0	**96.4**
0.065–0.0792	31	92.3	99.8	97.3	1.56	97.2	1	64.4	100	98.0	6.43	98.5	0	**97.8**
0.0798–0.0946	31	75.4	98.4	96.0	4.46	96.0	0	77.0	100	98.0	5.38	98.3	0	**97.1**
0.0955–0.1181	31	80.9	99.9	96.2	3.56	96.5	0	25.5	100	93.5	16.15	94.2	0	**95.1**
0.1181–0.145	31	67.1	99.8	95.2	5.63	95.3	0	53.4	100	97.2	8.53	97.1	0	**96.2**
0.152–0.292	30	59.4	98.9	94.0	7.36	94.2	0	58.4	100	96.4	8.47	96.8	0	**95.4**

Likewise, we tested the independence of our algorithm from the average heart rate size using all data in each database. We assigned every data into one of 10 pre-divided equal-bandwidth heart rate intervals, starting from 40 to 160 or 197 beats per minute. After calculating the performance scores SE and PPV for each data, we obtained the several statistic indexes and the gross and overall performance scores for each group, and the T-test (α = 0.05) was also performed. Their results for two databases are shown in Tables 5 and 6. Fig 13C and 13D are the curves of the performance score versus average heart rate for two databases.

**Table 5:** Performance scores of challenge database grouped by average heart rate (HR).

Average HR (Beats/Min)	Num. of Records	SE (%)						PPV (%)						Score (%)
		Min	Max	Ave.	σ	Gross	H	Min	Max	Ave.	σ	Gross	H	
40–52*	1	98.7	98.7	98.7	0	98.7	-	98.4	98.4	98.4	0	98.4	-	**98.6**
52–64	11	80.8	98.9	91.0	6.56	91.7	1	73.2	98.7	90.9	8.79	92.7	1	**91.6**
64–76	17	67.7	99.6	91.0	10.07	90.7	1	69.6	100	93.4	9.62	92.9	0	**92.0**
76–88	26	37.6	100	94.6	12.71	95.8	0	40.1	100	96.1	11.98	97.5	0	96.0
88–100	45	86.2	100	98.4	3.01	98.3	1	94.7	100	99.5	0.83	99.5	1	98.9
100–112	50	85.0	100	98.6	3.03	98.4	1	82.1	100	99.3	2.53	99.3	1	**98.9**
112–124	27	77.8	100	97.2	5.13	96.5	0	94.1	100	99.4	1.17	99.4	1	**98.1**
124–136	18	63.4	100	97.4	8.50	97.5	0	55.7	100	97.3	10.39	97.2	0	**97.3**
136–148*	1	98.7	98.7	98.7	0	98.7	-	100	100	100	0	100	-	**99.3**
148–165	4	83.7	99.0	92.6	6.43	92.6	0	99.8	100	99.9	0.11	99.9	1	**96.2**

*: Unable to do T-test for the only one sample.

**Table 6:** Performance scores of MGH/MF waveform database grouped by average heart rate (HR).

Average HR (Beats/Min)	Num. of Records	SE (%)						PPV (%)						Score (%)
		Min	Max	Ave.	σ	Gross	H	Min	Max	Ave.	σ	Gross	H	
40–52	6	94.7	99.4	96.3	2.11	96.3	0	94.5	100	98.0	2.04	98.1	0	**97.2**
52–64	7	88.7	100	95.7	7.32	95.7	0	91.3	100	97.5	3.23	97.5	0	**96.6**
64–76	30	67.1	100	95.6	6.36	95.5	0	53.4	100	97.2	8.59	96.7	0	**96.2**
76–88	42	82.5	99.8	96.9	5.57	96.9	0	93.2	100	99.4	1.29	99.5	1	**98.2**
88–100	37	88.9	100	97.5	3.44	97.5	1	85.8	100	99.0	2.49	99.1	1	**98.3**
100–112	41	42.3	99.3	95.9	9.12	96.0	0	44.8	100	98.0	8.66	98.1	0	**97.0**
112–124	19	80.9	99.9	96.7	1.66	97.4	0	25.5	100	95.3	16.97	96.6	0	**96.5**
124–136	11	92.9	99.7	97.5	4.08	97.6	0	81.0	100	97.6	5.63	97.8	0	**97.6**
136–148	3	95.2	99.0	96.9	13.51	96.9	0	94.1	100	98.0	3.35	98.2	0	**97.5**
148–197	4	86.5	97.4	92.8	3.85	92.8	0	98.7	99.8	99.4	0.47	99.4	1	**96.1**

In the above tables, H represents the T-test result, H = 0 represents that the group and the whole database pass the T-test at the level of α = 0.05, and the *H*_0_ hypothesis is established, that is, we have sufficient confidence to say that there is no significant difference between the group and the population. Most of the H = 1 cases in the tables are caused by the concentration of the high scores (the average is high and the σ is small), and a small part is caused by the low scores caused by the poor signal in the sample (the average is low and the σ is large), which may be related to the fact that the data is not a standard normal distribution. Seemly, the scores are slightly reduced with the increase of HRV and average heart rate from the figures, but the T-test results show that this is not a significant trend. Therefore, generally speaking, the performance of our algorithm is not affected by the heart rate variability and average heart rate size.

**Fig 14:**
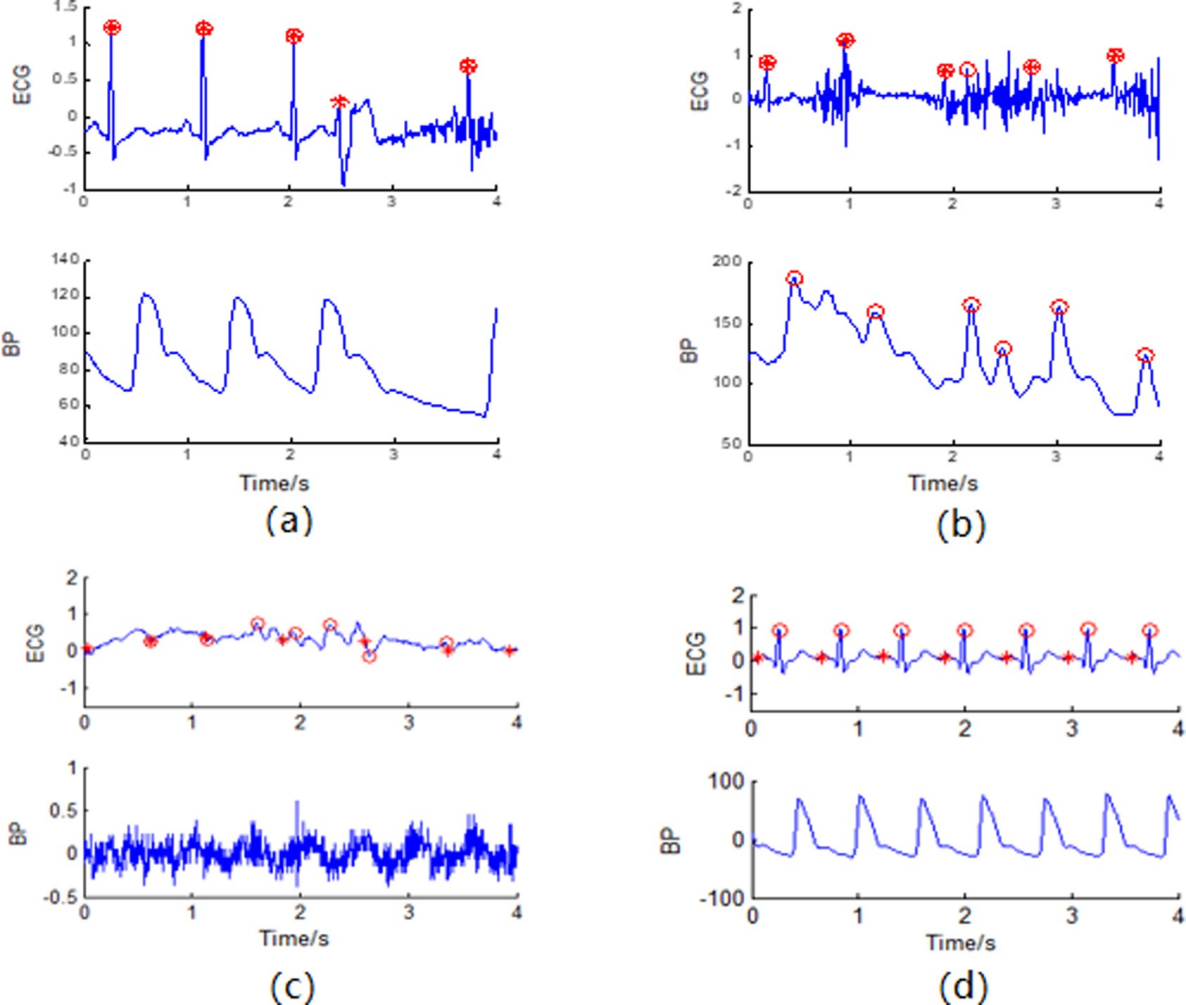
Cases of missed or redundant detections. (a) A missed mark in ECG; (b) A redundant mark in ECG; (c) Poor detecting results when the current ECG and BP segments are both of poor quality;(d)Inconsistent detecting results with the wrong official reference annotations, in which the * is the correct annotation, and the ◯ is the annotation using the proposed algorithm.

Compared with the official reference annotations, our algorithm still has some false marks or missed detections. Fig 14A shows a missed detection case when the quality of the BP is better than the ECG, and Fig 14B shows a case of redundant detection in which the BP marks include a redundant mark. Fig 14C shows a hard case that the current ECG and BP segments are both of poor quality (data mgh122 of MGH/MF).

Interesting, in our experiment, we found a data that its official reference annotations may have too large R-peak position mark errors, resulting in our algorithm’s low score for it, shown in Fig 14D (data mgh026 of MGH/MF database).

Above we discuss how to do a fusion to improve the R-wave peaks marking in this paper, actually, our core algorithm may allow for synchronous extraction of RR interval and systolic blood pressure, which can be used for spontaneous baroreflex testing. The synchronous (beat-by-beat) extraction of RR intervals and systolic blood pressure values in the testing is not trivial, as the two time series may get out of synchronization if R-waves are missed but the systolic blood pressure peaks are still detected (or vice versa). We are interested in doing some work in adjusting our algorithm to adapt to spontaneous baroreflex testing better in the future. Positively, we must not use fusion of the detected peak results of the two kinds of series any more.

## 5. Conclusion

In this article, we put forward an algorithm to detect R-wave peaks with the help of the BP signal under the condition that the ECG and BP signal are recorded simultaneously. Experimental results show that this algorithm can surely improve the detection rate and robustness of the ECG R-wave peak detection. The algorithm is robust to ECG pacemaker pulses and sudden changes in heart rate.
